# Accelerated long-term forgetting as a predictor of clinical onset in presymptomatic autosomal dominant Alzheimer’s disease

**DOI:** 10.1136/jnnp-2025-336750

**Published:** 2025-09-09

**Authors:** Nicholas Magill, Rachana Tank, Damien Ferguson, Duncan Alston, Antoinette O’Connor, Helen Rice, Kirsty Lu, Sebastian Crutch, Nick C Fox, Philip SJ Weston

**Affiliations:** 1 Dementia Research Centre, UCL Queen Square Institute of Neurology, London, UK; 2 London School of Hygiene & Tropical Medicine, London, UK; 3 UK Dementia Research Institute, London, England, UK

**Keywords:** ALZHEIMER'S DISEASE, COGNITION, DEMENTIA, MEMORY, COGNITIVE NEUROPSYCHOLOGY

## Abstract

**Background:**

In Alzheimer’s disease (AD), sensitive measures of cognitive decline prior to overt symptoms are urgently needed. Accelerated long-term forgetting (ALF), where new information is retained normally over conventional testing intervals but is then lost at an accelerated rate over the following days and weeks, has been identified cross-sectionally in presymptomatic autosomal dominant and sporadic AD cohorts. We aimed to assess whether ALF testing is predictive of proximity to future symptom onset.

**Methods:**

20 asymptomatic autosomal dominant AD mutation carriers who performed normally on standard cognitive testing underwent ALF assessment with (1) a list, (2) a story and (3) a visual figure, with the testing of 30-min recall and 7-day recall. Participants were followed up annually for a median of 7 years and assessed each time with the Clinical Dementia Rating (CDR) scale.

**Results:**

9/20 participants developed symptoms (CDR global>0) during follow-up. Those who became symptomatic had lower baseline ALF scores for both the list (progressors=30 (IQR, 30–36.4) and non-progressors=58.3 (IQR, 33–66.7), p=0.03) and story (progressors=58.8 (IQR, 44–66) and non-progressors=81.2 (IQR, 69.1–87.8), p<0.001). Story ALF (area under curve (AUC)=0.82) and list ALF (AUC=0.73) discriminated between those who did and did not develop symptoms.

**Conclusions:**

Severity of ALF is not only associated with the presence of AD pathology but also predictive of clinical onset, identifying those at the highest risk of imminent decline. ALF testing offers promise in aiding presymptomatic trial recruitment, as a presymptomatic cognitive endpoint and potentially as a screening tool in the wider population.

## Introduction

 In Alzheimer’s disease (AD), new disease-modifying therapies are likely to be most effective if given early, prior to significant neuronal loss, but we need to identify those people who are most likely to benefit.^1^ Identification of subtle cognitive changes occurring before symptom onset can help stratify those at the greatest risk of decline and assess treatment response.^2^ While it is widely accepted that episodic memory is the earliest cognitive domain affected in AD, conventional memory tests, which assess recall over intervals of 5–30 min, lack sensitivity to very early, presymptomatic changes.^3^

Autosomal dominant familial Alzheimer’s disease (ADAD), due to mutations in presenilin 1 (*PSEN1*), presenilin 2 or amyloid precursor protein (*APP*) genes, shares many features, both pathophysiologically and clinically, with the more common sporadic form, and allows prospective study of asymptomatic individuals with known AD pathology.^4^

Accelerated long-term forgetting (ALF) is a pattern of memory dysfunction whereby encoding and initial storage of new information occurs normally but is then lost at an accelerated rate over the following days and weeks and is thought to represent early breakdown in long-term consolidation.^5^ We previously found that ALF was a feature of presymptomatic ADAD in a group who were on average an estimated 7 years from symptom onset, had normal recall at 30 min and performed normally on all other cognitive tests.^6^ These findings have been replicated in multiple presymptomatic and sporadic AD cohorts.^7 8^ However, to date, studies of ALF have largely involved only cross-sectional group comparisons. To our knowledge, only one previous study, which assessed ALF in older people, has investigated its potential predictive value, but this was done in individuals for whom the presence or absence of AD pathology was unknown and with longitudinal follow-up over only 12 months.^9^ We investigated the value of ALF assessment in predicting future onset of clinical decline by undertaking extended longitudinal annual follow-up of asymptomatic ADAD mutation carriers following ALF testing.

## Methods

### Participants and procedures

20 asymptomatic ADAD mutation carriers were recruited between February 2015 and March 2016. 18 carried a *PSEN1* mutation and two an *APP* mutation. Participants were excluded if either they or their partner reported progressive cognitive symptoms or if they had any significant neurological or psychiatric condition that may influence their cognitive performance. All participants had a Clinical Dementia Rating (CDR) score of 0 (indicating no symptoms or functional impairment)^10^ and had performed normally on a comprehensive battery of standard cognitive testing, including an assessment of short interval episodic memory.^6^ ALF was tested using test materials from the Adult Memory and Information Processing Battery.^11^ Participants were assessed on initial learning, 30-min recall and 7-day recall of (1) a word list, (2) a short story and (3) a complex abstract visual figure using assessment procedures as described previously.^6^ For the word list, participants had to learn the material to a minimum required accuracy of 80% over a minimum of 4 and a maximum of 10 trials. For the story, the minimum required accuracy was 80%, over a minimum of two and a maximum of 10 trials. There was no minimum required learning score for the visual figure. The ALF score was calculated by dividing the 7-day recall score by the 30-min recall score and converting to a percentage, with higher scores representing better long-term retention. The initial testing, for learning and 30 min recall, was done in-person, with the 7-day testing done remotely. Participants were not told that the memory tests would be repeated 7 days following the initial testing. Participants underwent follow-up clinical assessment on an approximately annual basis, including completion of the CDR.

The research was approved by the Queen Square Ethics Committee. All participants provided informed consent.

### Statistical analysis

We compared ALF test scores (list, story and figure) of those who became symptomatic (CDR global>0) during follow-up with those who remained asymptomatic (CDR global=0) using the Kruskal–Wallis rank sum test. We present Kaplan–Meier plots, using median splits of ALF list, story and figure scores, to illustrate the relationship between ALF assessment and time to symptom onset in the two groups (ie, above median vs below median). We right-censored follow-up times at the final assessment date for participants who remained asymptomatic. Receiver operating characteristic curves were generated using logistic regression models to quantify the ability of baseline ALF scores to discriminate between those who would and would not go on to develop symptoms during follow-up.

In order to determine whether any association between test performance and symptom onset was unique to ALF assessment, we also analysed scores from several other established cognitive tests, again comparing those who did and did not become symptomatic. The cognitive tests included covered domains previously found to be affected early in AD,^12 13^ including three of episodic memory (Recognition Memory Test (RMT) words, RMT faces and Camden Paired Associate Learning), three of executive function (Digit Symbol Substitution, Trail Making A and Trail Making B) and two of global intellectual function (Wechsler Abbreviated Scale of Intelligence (WASI) verbal IQ and WASI performance IQ).

## Results

**Figure 1:**
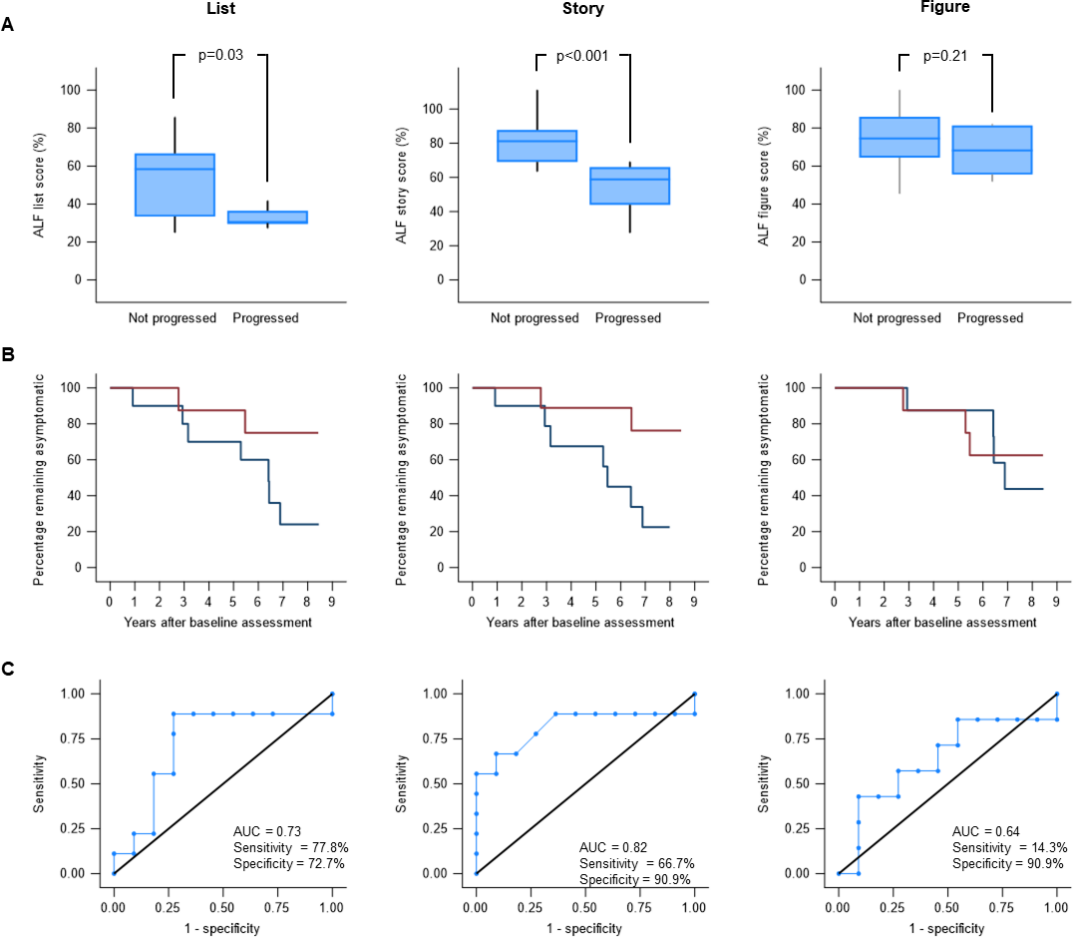
**(A**) Box plots showing the median ALF score (calculated as the percentage of information recalled at 30 min that is retained at 7 days) at baseline for list, story and figure tests, comparing those who remained asymptomatic and did not progress clinically with those who progressed to the symptomatic stage. (**B**) Kaplan–Meier curves showing the percentage of individuals who remain asymptomatic at each year of follow-up. The red lines represent individuals who scored above the median score on baseline list, story and figure ALF tests, while the blue lines represent those who scored below the median score. (**C**) Receiver operating characteristic curves for each of the three ALF scores showing the ability to discriminate between asymptomatic mutation carriers who do and do not go on to develop symptomatic disease during follow-up. For determining sensitivity and specificity, we used a cut-off probability of 0.5. ALF, accelerated long-term forgetting; AUC, area under the curve.

Baseline demographics and performance on both ALF assessment and other cognitive tests are shown in table 1. The median follow-up was 7 years. 9 participants developed symptoms (CDR global>0) during follow-up. Those who became symptomatic had significantly lower ALF scores at baseline for both the list (progressors=30 (IQR, 30–36.4) and non-progressors=58.3 (IQR, 33–66.7), p=0.03) and story (progressors=58.8 (IQR, 44–66), non-progressors=81.2 (IQR, 69.1–87.8), p<0.001) but not for the figure (progressors=68.2 (IQR, 55.6–81.3) and non-progressors=74.5 (IQR, 64.5–85.9) p=0.21) (figure 1A, table 1). While the list and story ALF scores (7 day/30 min) differed significantly between progressors and non-progressors, this was not the case for learning or 30-min recall scores. When splitting the participants based on the median ALF score, for both the list and story, the development of symptomatic disease occurred in seven individuals from the *below median* group and two individuals from the *above median* group (figure 1B). Both story ALF (area under curve (AUC)=0.82) and to a lesser extent list ALF (AUC=0.73) were able to discriminate between those mutation carriers who would and would not go onto develop symptoms, but this was not the case for figure ALF (AUC=0.64) (figure 1C).

**Table 1:** Summary of demographic and clinical measures: groups are compared using the Kruskal–Wallis rank sum test, with p values reported

	Progressors (n=9)	Non-progressors (n=11)	P value
Female (n (%))	4 (44.4%)	6 (54.6%)	
Age, years (mean (SD))	38.8 (3.9)	38.0 (7.2)	
Years in education (mean (SD))	13.9 (3.3)	14.2 (2.1)	
CDR global	0	0	>0.99
CDR Sum of Boxes	0	0	>0.99
MMSE at baseline	29 (29–30)	29 (29–30)	0.94
ALF list	30 (30–36.4)	58.3 (33–66.7)	0.03
ALF story	58.8 (44–66)	81.2 (69.1–87.8)	<0.001
ALF figure	68.2 (55.6–81.3)	74.5 (64.5–85.9)	0.21
WASI verbal IQ	106 (94–111)	103 (79–108)	0.32
WASI performance IQ	104 (103–114)	103 (100–117)	0.99
RMT words	49 (48–50)	50 (49–50)	0.24
RMT faces	46 (45–49)	44 (40–45)	0.13
Camden PAL	19 (16–22)	18 (12–22)	0.78
Digit Symbol Substitution Test	62.5 (52.5–72.5)	58.5 (52–61)	0.40
Trail Making A	19 (18.5–28.5)	28 (20–33)	0.17
Trail Making B	44 (37.5–68.5)	57 (43–67)	0.32

‘Progressors’ refer to those who developed cognitive symptoms during the follow-up period, while ‘non-progressors’ are those who remained asymptomatic. For all cognitive test scores, the values shown represent the median and IQR.

CDR, Clinical Dementia Rating; MMSE, Mini-Mental State Examination; PAL, Paired Associate Learning; RMT, Recognition Memory Test; WASI, Wechsler Abbreviated Scale of Intelligence.

No differences were found between those who did become symptomatic and those who did not become symptomatic for any of the other more conventional cognitive assessments (table 1).

## Discussion

In a group of asymptomatic ADAD mutation carriers who performed normally across baseline standard cognitive tests, we found that lower baseline verbal ALF scores were significantly associated with an increased risk of developing progressive clinical symptoms during the following 7 years. Those who went on to develop symptomatic disease had lower baseline ALF scores for both list and story recall, with story scores showing almost no overlap between the two groups. No similar association was found for any of the other cognitive tests of memory or other domains included in our standard testing battery.

Assessing long-term forgetting in asymptomatic people has previously identified cross-sectional group differences between those with and without AD pathology.^6^,^8^ It has also been found to have some predictive value of progression over a 12-month period in asymptomatic older people (although with unknown presence or absence of amyloid-β).^9^ Here, we extend on previous work by showing for the first time, over an extended follow-up period, that the severity of ALF is not only associated with the presence or absence of AD pathology but also predictive of proximity to clinical disease onset in people with known AD pathology, several years before symptoms develop. ALF testing therefore not only discriminates *between* groups (ie, with AD pathology vs without AD pathology) but can also discriminate *within* the AD group, identifying which people with presymptomatic AD are at the highest risk of decline.

While we can detect amyloid positivity through biomarkers, some amyloid positive individuals will be over 15 years from clinical onset and therefore may stand to gain less from intervention with disease-modifying treatments that have significant risk profiles.^14^ Additional markers of proximity to onset able to identify those most at risk of imminent decline are therefore needed to inform treatment decisions, with ALF testing providing a cheap and scalable potential solution. In the future, a combination of an accessible amyloid biomarker, such as emerging pTau217 blood tests,^15^ with an indicator of proximity to onset such as ALF assessment, could enable effective screening and treatment stratification.

As the field shifts to performing AD trials earlier, a step change is required in how we approach cognitive assessment in the period before symptoms. While still widely used in clinical trials, endpoints such as the CDR Sum of Boxes^10^ were designed to detect changes only after symptoms have developed and hence show very little dynamic change until that point. Moreover, in the current study, we also used several more conventional cognitive testing approaches, including those of memory, executive function and global intelligence, none of which showed an association with future symptom onset. Developing improved assessment tools for use in presymptomatic AD, able to detect earlier changes, is an area of high importance. However, efforts to date have tended to use lengthy combinations of multiple tests that were not originally designed for use during the presymptomatic period.^16 17^

While the results of verbal ALF assessments—both list and story—were associated with future symptom onset, this was not the case for long-term recall of a visual figure. This finding is consistent with long-term memory in the verbal domain being affected earliest in AD^13^ and with our previous work showing the cross-sectional discriminatory power of verbal ALF to be higher than visual.^6^ However, an alternative explanation is that the visual figure used did not represent a sensitive enough assessment of forgetting in the visual domain, with recent work showing that sensitivity can be improved by selectively assessing recall of more salient features.^18^

Our study has limitations. The sample size is relatively small, primarily because of the relative rarity of ADAD, although it was still sufficient to identify group differences, thereby highlighting the strong effect size. Replication in both familial and sporadic presymptomatic AD cohorts will be important. A potential practical limitation of our ALF testing paradigm, and its widespread implementation clinically and in trials, is its relatively high resource demands, with assessment required on 2 days 1 week apart. The development of automated digital testing paradigms could help to address this issue.

In summary, we have shown that the assessment of verbal ALF may provide a means of identifying individuals with presymptomatic AD pathology who are at the highest risk of developing cognitive symptoms and clinical decline in the coming years. ALF testing offers promise in aiding presymptomatic clinical trial recruitment as a presymptomatic cognitive endpoint and ultimately as a cognitive screening tool in the wider population.

## References

[1] Aisen PS, Jimenez-Maggiora GA, Rafii MS (2022). Early-stage Alzheimer disease: getting trial-ready. Nat Rev Neurol.

[2] Mortamais M, Ash JA, Harrison J (2017). Detecting cognitive changes in preclinical Alzheimer’s disease: A review of its feasibility. Alzheimers Dement.

[3] Sperling RA, Aisen PS, Beckett LA (2011). Toward defining the preclinical stages of Alzheimer’s disease: Recommendations from the National Institute on Aging‐Alzheimer’s Association workgroups on diagnostic guidelines for Alzheimer’s disease. Alzheimers Dement.

[4] Bateman RJ, Aisen PS, De Strooper B (2010). Autosomal-dominant Alzheimer’s disease: a review and proposal for the prevention of Alzheimer’s disease. *Alzheimers Res Ther*.

[5] Rodini M, De Simone MS, Caltagirone C (2022). Accelerated long-term forgetting in neurodegenerative disorders: A systematic review of the literature. Neurosci Biobehav Rev.

[6] Weston PSJ, Nicholas JM, Henley SMD (2018). Accelerated long-term forgetting in presymptomatic autosomal dominant Alzheimer’s disease: a cross-sectional study. Lancet Neurol.

[7] Zimmermann JF, Butler CR (2018). Accelerated long-term forgetting in asymptomatic APOE ε4 carriers. Lancet Neurol.

[8] Tort-Merino A, Valech N, Laine M (2021). Accelerated long-term forgetting in individuals with subjective cognitive decline and amyloid-β positivity. Int J Geriatr Psychiatry.

[9] Wearn AR, Saunders-Jennings E, Nurdal V (2020). Accelerated long-term forgetting in healthy older adults predicts cognitive decline over 1 year. *Alz Res Therapy*.

[10] Morris JC (1997). Clinical dementia rating: a reliable and valid diagnostic and staging measure for dementia of the Alzheimer type. Int Psychogeriatr.

[11] Coughlan AK, Hollows SE (1985). The adult memory and information processing battery (AMIPB): test manual.

[12] Bondi MW, Jak AJ, Delano-Wood L (2008). Neuropsychological contributions to the early identification of Alzheimer’s disease. Neuropsychol Rev.

[13] Fox NC, Warrington EK, Seiffer AL (1998). Presymptomatic cognitive deficits in individuals at risk of familial Alzheimer’s disease. A longitudinal prospective study. Brain (Bacau).

[14] Dubois B, Epelbaum S, Nyasse F (2018). Cognitive and neuroimaging features and brain β-amyloidosis in individuals at risk of Alzheimer’s disease (INSIGHT-preAD): a longitudinal observational study. Lancet Neurol.

[15] Janelidze S, Barthélemy NR, Salvadó G (2024). Plasma Phosphorylated Tau 217 and Aβ42/40 to Predict Early Brain Aβ Accumulation in People Without Cognitive Impairment. JAMA Neurol.

[16] Donohue MC, Sperling RA, Salmon DP (2014). The preclinical Alzheimer cognitive composite: measuring amyloid-related decline. JAMA Neurol.

[17] Langbaum JB, Ellison NN, Caputo A (2020). The Alzheimer’s Prevention Initiative Composite Cognitive Test: a practical measure for tracking cognitive decline in preclinical Alzheimer’s disease. *Alz Res Therapy*.

[18] Lu K, Baker J, Nicholas JM (2025). Associations between accelerated forgetting, amyloid deposition and brain atrophy in older adults. Brain (Bacau).

